# Metabolite profile in hereditary spastic paraplegia analyzed using magnetic resonance spectroscopy: a cross-sectional analysis in a longitudinal study

**DOI:** 10.3389/fnins.2024.1416093

**Published:** 2024-08-13

**Authors:** Domenico Montanaro, Marinela Vavla, Francesca Frijia, Alessio Coi, Alessandra Baratto, Rosa Pasquariello, Cristina Stefan, Andrea Martinuzzi

**Affiliations:** ^1^U.O. Dipartimentale e Servizio Autonomo di Risonanza Magnetica, Dipartimento di Neuroscienze dell’Età Evolutiva, IRCCS Fondazione Stella Maris, Pisa, Italy; ^2^Child and Adolescent Neuropsychiatric Unit, Department of Women’s and Children’s Health, University Hospital of Padua, Padova, Italy; ^3^Department of Neurorehabilitation, IRCCS E. Medea Scientific Institute, Conegliano, Italy; ^4^Bioengineering Unit, Fondazione Toscana G. Monasterio, Pisa, Italy; ^5^Unit of Epidemiology of Rare Diseases and Congenital Anomalies, Institute of Clinical Physiology, National Research Council, Pisa, Italy; ^6^Department of Radiology, S. Maria dei Battuti Hospital- Conegliano, Treviso, Italy

**Keywords:** hereditary spastic paraplegias (HSP), magnetic resonance spectroscopy (MRS), cross sectional analysisis, longitudinal analysis, pre-frontal, SPRS, conditional inference tree method

## Abstract

**Background:**

Hereditary Spastic Paraplegias (HSP) are genetic neurodegenerative disorders affecting the corticospinal tract. No established neuroimaging biomarker is associated with this condition.

**Methods:**

A total of 46 patients affected by HSP, genetically and clinically evaluated and tested with SPRS scores, and 46 healthy controls (HC) matched by age and gender underwent a single-voxel Magnetic Resonance Spectroscopy sampling (MRS) of bilateral pre-central and pre-frontal regions. MRS data were analyzed cross-sectionally (at T_0_ and T_1_) and longitudinally (T_0_ vs. T_1_).

**Results:**

Statistically significant data showed that T_0_ mI/Cr in the pre-central areas of HSP patients was higher than in HC. In the left (L) pre-central area, NAA/Cr was significantly lower in HSP than in HC. In the right (R) pre-frontal area, NAA/Cr was significantly lower in HSP patients than in HC. HSP SPG4 subjects had significantly lower Cho/Cr concentrations in the L pre-central area compared to HC. Among the HSP subjects, non-SPG4 patients had significantly higher mI/Cr in the L pre-central area compared to SPG4 patients. In the R pre-frontal area, NAA/Cr was reduced, and ml/Cr was higher in non-SPG4 patients compared to SPG4 patients. Comparing “pure” and “complex” forms, NAA/Cr was higher in pHSP than in cHSP in the R pre-central and R pre-frontal areas. The longitudinal analysis, which involved fewer patients (*n* = 30), showed an increase in mI/Cr concentration in the L pre-frontal area among HSP subjects with respect to baseline. The patients had significantly higher SPRS scores at follow-up, with a significant positive correlation between SPRS scores and mI/Cr in the L pre-central area, while in bilateral pre-frontal areas, lower SPRS scores corresponded to higher NAA/Cr concentrations. To explore the discriminating power of MRS in correctly identifying HSP and controls, an inference tree methodology classified HSP subjects and controls with an overall accuracy of 73.9%, a sensitivity of 87.0%, and a specificity of 60.9%.

**Conclusion:**

This pilot study indicates that brain MRS is a valuable approach that could potentially serve as an objective biomarker in HSP.

## 1 Introduction

Hereditary spastic paraplegia (HSP) encompasses a large group of rare, clinically, and genetically composite conditions (prevalence 1-5 per 100,000, ORPHAcode: 685). Key clinical manifestations include lower extremity spasticity, urinary urgency, and impairment of lower extremity vibratory sensation. According to Harding (1983) criteria, when the neurological impairments are limited to these symptoms, the phenotype can be classified as “pure HSP” (or uncomplicated - pHSP) (Ferrara et al., 2024). In cases where other neurological disturbances (such as epilepsy, extrapyramidal signs, neuropathy, cerebellar ataxia, dysarthria, and peripheral neuropathy), intellectual disability, and systemic symptoms (bladder dysfunction, defecatory dysfunction, pes cavus, and visual deficit) are present, the phenotype is defined as “complex” (or complicated - cHSP). In cHSP, compared to pHSP, the age of onset is younger, males have a significantly higher severity, and cognitive function is the most frequent (Cui et al., 2018; Boutry et al., 2019).

Historically, the histological hallmark of HSP is pyramidal tract damage secondary to progressive “dying back” corticospinal tract (CST) degeneration due to altered intracellular axonal transport within long fibers, likely caused by a generalized failure of the nerve cells to maintain the vitality of long axons (Behan and Maia, 1974). In both pure and complicated HSP, quantitative methods detecting altered patterns distributed across the central nervous system suggest a more complex neurodegenerative process involving extra-motor regions (Agosta et al., 2015; Martinuzzi et al., 2016). This could include, for example, the pre-frontal cerebral areas, which are associated with clinical cognitive aspects and motor associative functions. This has led some authors to focus specific studies on that area (Erichsen et al., 2009; Liguori et al., 2014).

The mechanisms underlying the degeneration of distal portions of the CST are still unknown. However, the identification of numerous causal genes correlated with the functions of their products suggests that alterations to intracellular trafficking may be the most frequent cause. These alterations include active axonal transport, activities of the endolysosomal system, organelle shaping, and lipid metabolism (Boutry et al., 2019). In some forms, gene products are known to be involved in multiple pathways, such as myelination, mitochondrial functions, and axon guidance (Mackay-Sim, 2021).

Currently, HSPs are classified according to the mode of inheritance (autosomal, recessive, or X-linked) and their specific chromosomal gene or locus, designated as “SPG” (SPastic parapleGia or Spastic Paraplegia Gating), followed by a progressive number. The designation of SPG has been given to more than 90 clinical-genetic forms of HSP, and more than 25 genes causing HSP have not been assigned an SPG designation (“non-SPG” genes) (Mahale et al., 2023). When suspecting HSP, a family history or consanguinity supports the possibility of a hereditary condition, but its absence should not dissuade the clinician from the diagnosis. In this case, it is fundamental to exclude acquired myelopathies mimicking HSP (human T-lymphotropic virus-related myelopathy, primary progressive multiple sclerosis, vitamin B12 deficiency, copper deficiency, spinal cord tumors or malformations, and degenerative disorders of the spine), for which magnetic resonance imaging (MRI) plays a vital role (Faber et al., 2017).

Spinal cord MRI is not particularly indicative of HSP (da Graça et al., 2018). Instead, routine brain MRIs reveal some signs specific to certain genetic HSP subtypes. These could be helpful in cases of childhood or adolescent onset and can direct more accurate diagnostic investigations when HSP might be mistaken for cerebral palsy or other neurodevelopmental disorders (Dosi et al., 2021). A thin corpus callosum (TCC) is the most classic MRI finding suggestive of HSP. This sign led to the creation of a group of patients (HSP-TCC) in which SPG11 is the more frequent HSP type, but it may also be present in SPG52, where the splenium is mostly affected, and in SPG30 and SPG54, with the body and splenium most involved. Only in SPG11 a progression of the thinning is reported (Dosi et al., 2021).

Other MR signs described in HSP include the “ear-of-the-lynx sign,” corresponding to an abnormality of the forceps minor of the corpus callosum, appearing hyperintense on T2-FLAIR-weighted images and hypointense on T1-weighted images. Others sign, though very variable and not indicative of HSP (da Graça et al., 2018), include hydrocephalus in SPG1 and SPG4 patients; leukoencephalopathy, a much more evident feature in Merzbacher disease but also present in SPG2; multifocal areas of T2 white matter hyperintensities, sometimes resembling multiple sclerosis, in SPG5 and SPG35; basal ganglia iron deposition appearing hypointense on T2, T2,^*^ or Susceptibility Weighted Images (SWI) in SPG28, SPG35, and SPG43; basal ganglia abnormalities in SPG7 with cerebellar atrophy.

To date, the treatments for HSP include antispasticity drugs, botulinum toxin, and physiotherapy. Clinical trials on HSP patients have yielded only a few positive results (Gumeni et al., 2021). In this context, outcome evaluations and biomarkers that can measure the efficacy of therapeutic interventions in clinical trials are urgently needed (Siow et al., 2023). The current 83 outcome measures are heterogeneous and inconsistent (Siow et al., 2023).

For a diagnostic evaluation, metabolic profiling of cerebrospinal fluid (CSF) or *ex vivo* metabolic profiling of the brain tissue could be able to characterize the neurochemical status of the central nervous system (CNS), but they are justified only for diagnostic purposes and are not ideal for repeated follow-up or screening in the general population (McKay and Tkáč, 2016).

In the last three decades, scientific reports using MRI techniques have investigated structural and functional changes in HSP (Agosta et al., 2009). These techniques were initially called “advanced,” but the term “quantitative MRI” is sometimes preferred. They include structural imaging (voxel-based morphometry and cortical thickness), diffusion tensor imaging (DTI, anisotropy), spectroscopy (MRS), iron-sensitive sequences (T2^*^ and SWI), and functional MR imaging (fMRI) (Pioro, 2000, 2002; Mazón et al., 2018).

Among these techniques, proton MRS (1H-MRS) provides biochemical information on metabolites in the CNS. MRS is a neuroimaging method used to investigate *in vivo* brain metabolites, aiming to unveil the underlying biochemical mechanisms and monitor disease processes and treatment responses. Conventional “proton” MRI essentially represents the spatial distribution of water. When the MR signal derived from water is suppressed, it is possible to highlight the protons in metabolites that are much less abundant than water (Kalra and Arnold, 2004). The MR spectrum is represented by a graph in which the x-axis denotes the different chemical frequencies of the full sample in parts per million (ppm), while the y-axis relates to their concentrations. Spectra are obtained either from one selected brain region in the case of single-voxel spectroscopy or from multiple voxels in the case of MR spectroscopic imaging (Mazón et al., 2018).

Among the metabolites revealed with 1H-MRS, creatine (Cr) is usually unaffected in non-acute, non-destructive pathologies. Therefore, it provides a convenient internal reference that can also reduce influences deriving from magnetic field inhomogeneity, partial volume effects, and metabolite relaxation properties. Other metabolites revealed in MRS sampling are often reported as ratio relative to Cr. In some metabolic disorders (e.g., mitochondrial diseases) and tumors (e.g., medulloblastoma, meningioma, and lymphoma), Cr can vary, consequently altering the ratios referred to it. To overcome this effect, “absolute” quantitation of metabolite concentrations is more desirable than ratios, posing challenges to precision and accuracy. The most frequently employed strategy is to acquire the referring water signal of the brain (Pizzini et al., 2003). Unfortunately, the exact water content (~45 mol/L for normal gray matter and ~ 40 mol/L for normal white matter) is often unknown, especially in the neonatal brain and in most brain pathologies (Liserre et al., 2021). Since MRS single-voxel techniques are easy to apply in routine clinical examinations, most studies in HSP are based on significant intergroup differences, so these techniques continue to be in use (Kalra and Arnold, 2004; Kalra, 2019).

Among the more than 30 metabolites identifiable with 1H-MRS (Mazón et al., 2018), the most commonly investigated are as follows:

- *N-acetyl aspartate (NAA)*: A marker of neuronal integrity, low in a healthy developing newborn and increasing rapidly with brain maturation, becoming the dominant peak by 6 months of age, reaching its maximum at approximately 10–15 years.
- *Choline (Cho)*: A marker of cell membrane turnover, dependent on phosphorylcholine (~0.6 mM) and glycerophosphorylcholine (GPC, ~1 mM) levels, which can decrease during and after the postnatal period until the age of 3 years and can be elevated in cases of increased membrane turnover, such as in tumors, acute ischemia, demyelination, inflammation, and gliosis.
- *Creatine (Cr)*: A marker of energy metabolism, originating from the CH3 group of the molecule from free creatine (fCr) and phosphocreatine (PCr), is used to maintain ATP levels and is considered a marker of the potentially available energy, increasing rapidly before and around term.
- *Myo-inositol (mI)*: Described as an astroglial marker, which can change in response to alterations of membrane metabolism or damaged membrane, such as in gliomas or in some electrolyte alterations, particularly in hyponatremia and hepatic encephalopathy, usually high in the neonatal brain and decreasing in the first 24 months of life.
- *Lactate (Lac)*: A product of anaerobic glycolysis, increasing when oxidation in the Krebs cycle is impaired (hypoxia or mitochondrial diseases), representing an indicator of anaerobic metabolism.
- *Lipids (Lip)*: Accumulated as catabolites of brain damage, protons of the CH3 (methyl) and CH2 (methylene) groups, having broad peaks and overlapping with other macromolecules, normally visible in the myelinating brain of the first years of life, and considered a signal of cell membrane breakdown, particularly in necrosis and severe brain injury. They can sometimes be confused with fluctuating baselines.
- *Glutamate and glutamine (Glx)*: At 1.5 T, they almost overlapped and were collectively termed “Glx.” Glx is a marker of the glutamatergic neurotransmitter system, higher in astrocytes, increased in hypoxic states, and hyperammonemia disorders.
- *γ-Aminobutyric acid (GABA)*: An inhibitory neurotransmitter identifiable only in particular conditions and with dedicated spectral methods.

It would be rather naïve to expect that concentration changes in a just these few brain metabolites would be capable of probing the complex network of brain neurochemical processes. Moreover, reports on the MRS methodology applied in HSP are inhomogeneous, particularly due to technical aspects (such as different MR field strengths, technical parameters, and brain regions sampled) and the inclusion of mixed SPG-type patients in the same study, which does not allow for the comparison of results (Vavla et al., 2020). Different clinical phenotypes, genotypes, or stages of disease progression of the same neurodegenerative disease can also yield different MRS data, with findings showing either near-normal levels of metabolites or decreased or increased levels in the same SPG group (Pizzini et al., 2003).

The present study was set up to investigate: (a) whether HSP leads to biochemical MRS changes that allow discriminating a large cohort of HSP patients from matched healthy controls (HC) by sampling the pre-central areas as specific control of motor functions, and the pre-frontal areas, as motor associative and/or cognitive roles (biomarker of diagnosis) and (b) in a reduced group of the same HSP patients and healthy controls, longitudinally brain metabolite changes (a biomarker of disease progression) using the identical MRS methodology.

## 2 Materials and methods

### 2.1 Patients

The patients and HCs included in the current study were partially already reported by the authors of the present study (Montanaro et al., 2020). The sample of this study consisted of 46 HSP patients (mean age 41.5 ± 15.9 years) who attended the Medea Scientific Institute in Conegliano (TV), Italy, and 46 healthy controls, matched by age and gender (Table 1).

**Table 1:** Demographic, clinical, and genetic data of HSP patients and healthy control group.

	HSP patients *N* = 46	Healthy controls *N* = 46
Age at first MRI (years)	41.5 ± 15.9	42.2 ± 12.2
Gender (female)	25 F, 21 M	32 F, 14 M
Disease duration (years)	27 ± 20	
Disease onset (years)	15 ± 13	
Spastic gate loci		
SPG4	18	
SPG5	5	
SPG11	2	
SPG30	1	
SPG3	6	
SPG8	2	
SPG7	3	
SPG72	2	
SPG10	1	
SPG31	1	
SPG?	5	
Pure/complicated	27/14	

All the patients with HSP underwent molecular genetic studies. According to the genetic results, the 46 HSP patients were subdivided into SPG4 (18 pts), SPG3 (6 pts), SPG5 (5 pts), SPG7 (3 pts), SPG11 (2 pts), SPG8 (2 pts), SPG72 (2 pts), SPG10 (1 pt), SPG30 (1 pt), and SPG31 (1 pt). HSP patients were also subdivided into “pure” (27 pts) and “complicated” (14 pts) based on the clinical presentation. Five patients had undetermined SPG typing.

### 2.2 Clinical evaluations

Disease severity measures (DD) were assessed with various clinical tests, such as the modified Ashworth scale, the 6-min walking test (6MWT), lower limb deep tendon reflexes (LL DTR) grading (0–4), LL muscle strength assessed with the Medical Research Council (MRC) megascore, distal muscle wasting, cognitive functioning assessed with the WAIS-R, and the Spastic Paraplegia Rating Scale (SPRS) (Schüle et al., 2006). The latter measure was used for statistical analysis.

### 2.3 MRS protocol

All subjects underwent an MRI study with 1.5 T equipment (Philips Achieva 2.5 XR, Royal Philips Healthcare, Eindhoven, NL) at baseline (T_0_) and follow-up (T_1_), with a mean interval between the two examinations of 2.42 ± 0.81 years.

**Figure 1:**
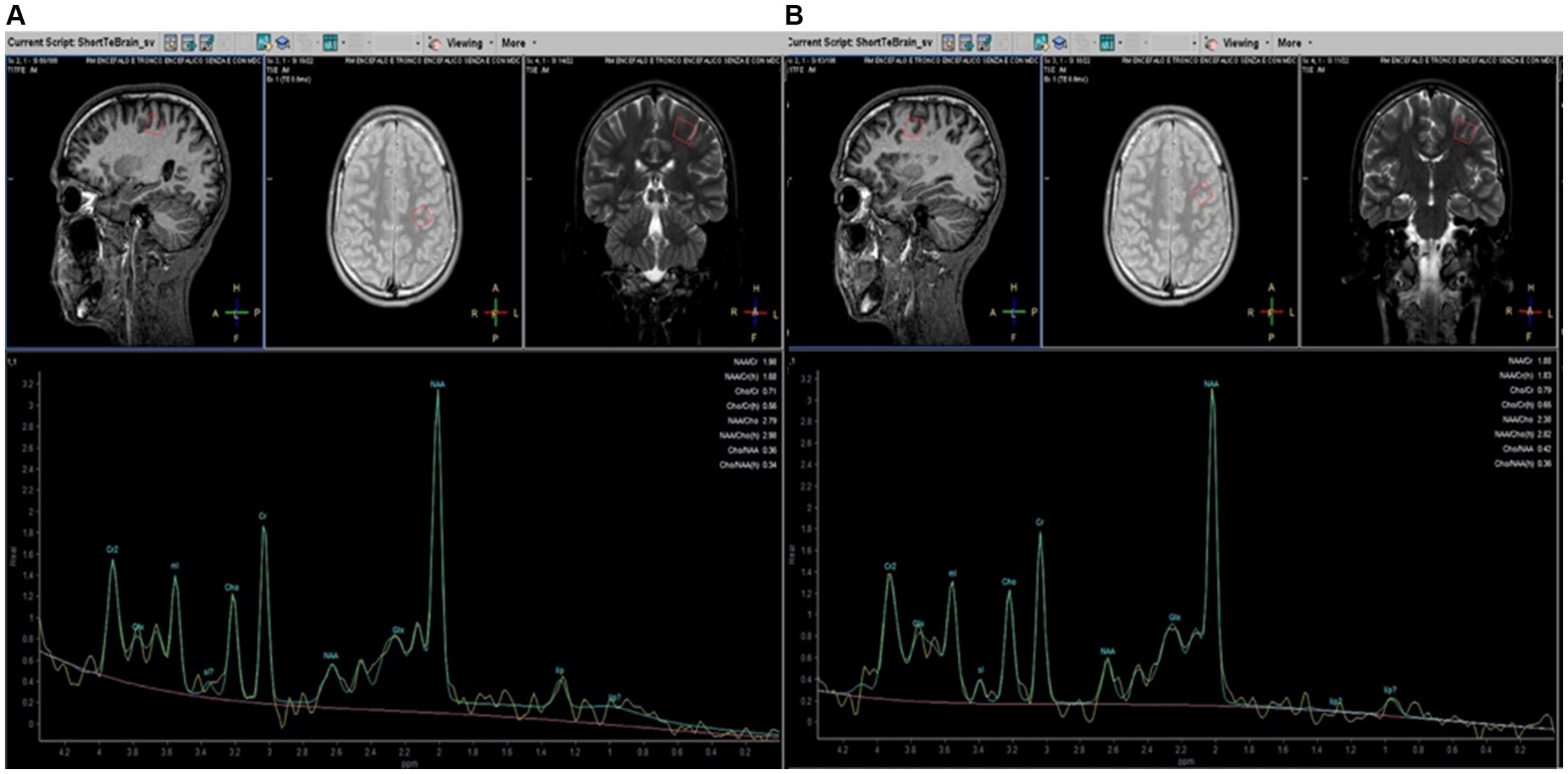
Parasagittal, axial, and coronal brain images (on the top) illustrating placement of MRS voxels (red line. 15 × 15 × 15 mm) in the pre-central **(A)** and pre-frontal **(B)** left cerebral hemispheres in patients with HSP. MRS spectra profiles are represented at the bottom of the corresponding localization. The peaks are automatically identified using dedicated software (Spectro View Software, Philips Healthcare, NL). The main metabolites are indicated on each peak.

MRI examinations included a standard protocol to exclude other pathologies (T2, T2-FLAIR, and T1 weighted images). The findings were partially described in a previous study (Montanaro et al., 2020) and did not differ from other reports (Dosi et al., 2021). T1-weighted 3D-MPRAGE or T2-weighted images were used to manually place each voxel of the spectroscopic sampling (voxel size 15 × 15 × 15 mm), centered bilaterally on the projection of the knob in the pre-central area, considered the region of the hand motor area, and anteriorly in the pre-frontal cortex, expression of motor and extra-motor associative areas (Figure 1), under the supervision of two experienced neuroradiologists (AB and DM). Due to the technical elasticity of the machine, the positioning of each voxel included more gray matter than white matter (not calculated) and avoided incorporating cerebrospinal fluid spaces and the bone. Single-voxel 1H-spectra were sampled with a total of 4 for each patient in a cross-sectional (T_0_) and longitudinal study (T_1_). In all the patients and HCs, the sampled areas had no MRI signal or morphological alterations.

We used a PRESS sequence with a short TE (TR = 2,000 ms, TE = 35 ms) that allows increased SNR compared to long TE and is less sensitive to T2 contributions to the signal (Yousem et al., 1991). Data were processed using Spectro View Software (Philips Healthcare, NL), measuring peaks and areas under the curves as ratios referred to as Cr, considered normative inter-subject units to define a peak table for each metabolite. The main brain metabolites detectable with 1H-MRS were analyzed: NAA (precessing at 2 ppm), Cho (3.2 ppm), Cr (3.07 ppm), and mI (3.5 ppm). Moreover, it is possible to increase sensitivity to coupled spins (i.e., from protons of the -CH_2_- groups) that can characterize molecules such as amino acids (2–3 ppm range) and lipids. Among lipids, a couple of peaks between 0.9 and 1.30 ppm are automatically related to lipids’ different peaks, as demonstrated by the vendor’s specifics to different methyl ligands. As for other studies with MRS in neurodegenerative diseases, spectra were excluded from further analysis if visual inspection showed data corruption (Dey et al., 2023). No attempt was made to provide absolute quantification of our 1H-MRSI data for the complexity and inaccuracies associated with deriving absolute concentrations from *in vivo* 1H-MRSI data, particularly using 1.5 T equipment (Stromillo et al., 2011).

### 2.4 Statistical analysis

MRI data were analyzed cross-sectionally at T_0_ and longitudinally (T_0_ vs. T_1_).

Datasets were discarded based on the quality of the single acquisitions and the presence of movement artifacts. In each investigated area (pre-central and pre-frontal, right and left), the Student’s *t*-test was used for data showing a parametric distribution. The nonparametric Wilcoxon test was used for continuous variables to evaluate differences in metabolite levels between the following: (I) HSP vs. HC; (II) HSP subjects with the genetic profile SPG4 vs. HC; (III) Genetic profile SPG4 vs. HSP with different genetic profiles; (IV) “Pure” vs. “Complex”; (V) “Pure” vs. HC; (VI) “Complex” vs. HC; (VII) Correlation with SPRS and disease duration (DD) at T_0_ in HSP.

Multilinear regression models were also adjusted for sex and age at T_0_ whenever Students’ *t*-tests showed statistically significant differences.

A paired Students’ *t*-test was used to assess significant differences in metabolites between baseline (T_0_) and follow-up (T_1_) in HSP and HC subjects (longitudinal analysis).

The effect size for comparing the difference between the means of the two groups was calculated using Cohen’s method. In the linear regression models used to assess the correlation between metabolites and SPRS and between metabolites and DD at T_0_, the effect size was calculated using eta-squared estimates with a 95% confidence interval (*p* = 0.05).

The longitudinal analysis also compared differences between controls and HSP subjects. In doing so, as already done by other authors (Piccinin et al., 2020), we subtracted the measures obtained at T_1_ from those obtained at T_0_ for each patient and each control. Then, we assessed the group differences (patients vs. controls) by applying an analysis of variance, adjusting for sex and age.

Mean values with a standard deviation (±SD) were reported in the text. In all analyses, a two-sided *p*-value of <0.05 was considered statistically significant. Statistical analyses were conducted using STATA software (Statistical Software for Data Science, 2024).

Conditional inference trees, a function of recursive partitioning for continuous variables, were also used to identify a subset of metabolites characterizing HSP subjects and controls (Hothorn et al., 2006). Conditional inference trees were performed using the R statistical package (R: The R Project for Statistical Computing, 2024).

### 2.5 Ethics statement

The study was approved by the competent Ethics Committee (# 06/2020CESC) and was conducted in accordance with the ethical standards of the Declaration of Helsinki (1964). Control subjects were adults enrolled as volunteers or normal developing children from the routine clinical MRI waiting list, assigned to a brain MRI examination due to unrelated causes (e.g., headache or vertigo). None of the controls had a neurological or psychiatric diagnosis.

All adult participants and their parents or legal guardians provided written informed consent before inclusion in the study. All related documents were collected and stored by the clinical investigators at the center (MV, AM), according to IRB guidelines.

## 3 Results

### 3.1 Cross-sectional analysis

The statistically significant differences in metabolites between the investigated groups are reported in Table 2. At T_0,_ the values of mI/Cr of the pre-central areas of HSP subjects (L 0.64 ± 0.12; *R* = 0.62 ± 0.22) were significantly higher than in HC (L 0.562 ± 0.09; R 0.56 ± 0.08) (L pre-central *p* = 0.001, R pre-central *p* = 0.04, after adjustment for sex and age).

**Table 2:** Cross-sectional at T_0_ MRS results.

	Cross sectional analysis—comparison between groups					
Comparison	Metabolites ratio	Area	HSP patients	Healthy controls	*p*-value	Effect size
HSP vs. HC	mI/Cr	L Pre-central	0.64 ± 0.12	0.56 ± 0.09	0.001	−0.73
HSP vs. HC	mI/Cr	R Pre-central	0.62 ± 0.22	0.56 ± 0.08	0.040^*^	−0.39
HSP vs. HC	NAA/Cr	L Pre-central	1.77 ± 0.20	1.85 ± 0.18	0.020	0.42
HSP vs. HC	NAA/Cr	R Pre-frontal	1.72 ± 0.18	1.83 ± 0.18	0.020	0.68
HSP SPG4 vs. HC	Cho/Cr	L Pre-central	0.71 ± 0.10	0.79 ± 0.15	0.012	0.60
SPG4 vs. no-SPG4	mI/Cr	L Pre-central	SPG4: 0.58 ± 0.13 no-SPG4: 0.69 ± 0.11	–	0.011	0.90
SPG4 vs. no-SPG4	NAA/Cr	R Pre-frontal	SPG4: 2.08 ± 0.18 no-SPG4: 1.81 ± 0.18	–	0.014	−1.05
SPG4 vs. no-SPG4	mI/Cr	R Pre-frontal	SPG4: 0.57 ± 0.13 no-SPG4: 0.67 ± 0.11	–	0.03	0.84
pHSP vs. HC	Cho/Cr	L Pre-central	0.71 ± 0.11	0.79 ± 0.15	0.012	0.61
pHSP vs. HC	mI/Cr	L Pre-central	0.62 ± 0.13	0.56 ± 0.09	0.032	0.50
cHSP vs. HC	NAA/Cr	R Pre-central	1.83 ± 0.31	2.06 ± 0.24	0.001^*^	0.89
cHSP vs. HC	mI/Cr	L Pre-central	0.70 ± 0.11	0.56 ± 0.09	<0.001^*^	−1.36
cHSP vs. HC	NAA/Cr	R Pre-frontal	1.64 ± 0.16	1.84 ± 0.18	0.004	1.15
“Pure” HSP vs. “complex” HSP	NAA/Cr	R Pre-central	1.81 ± 0.20 1.63 ± 0.26	–	0.027	−0.78
“Pure” HSP vs. “complex” HSP	NAA/Cr	R Pre-frontal	p: 1.81 ± 0.14 c: 1.64 ± 0.16	–	0.021	−1.14

Cross sectional analysis—correlation with SPRS and DD					
Correlation	Metabolites ratio	Area	Coefficient	*p*-value	Effect size
With SPRS in HSP	mI/Cr	L Pre-central	0.004	0.032	0.11
With SPRS in HSP	NAA/Cr	R Pre-frontal	−0.009	0.014	0.20
With SPRS in HSP	NAA/Cr	L Pre-frontal	−0.012	0.005	0.35
With DD in HSP	Cho/Cr	L Pre-central	0.004	0.021	0.18

When data have a non-parametric distribution, for each metabolite both median with IQR and mean ± SD are reported. L, left; R, right. ^*^p-value referred to Wilcoxon rank sum test.

In the L pre-central area, the level of NAA/Cr was significantly lower in HSP subjects than in HC (1.77 ± 0.20 vs. 1.85 ± 0.18; *p* = 0.02, after adjustment for sex and age at T_0_).

Similarly, in the R pre-frontal area, NAA/Cr was significantly lower in HSP subjects than in HC subjects (1.72 ± 0.18 vs. 1.83 ± 0.18; *p* = 0.02, after adjustment for sex and age at T_0_).

Restricting the analysis only to HSP SPG4 subjects vs. HCs, there were significantly lower Cho/Cr concentrations in the L pre-central area in patients (0.71 ± 0.10 vs. 0.79 ± 0.15; *p* = 0.012, after adjustment for sex and age at T_0_).

The analysis comparing SPG4 vs. non-SPG4 among HSP subjects showed that in the L pre-central area, the measured levels of mI/Cr in subjects with non-SPG4 were significantly higher than those in subjects with SPG4 (0.58 ± 0.13 vs. 0.69 ± 0.11, *p* = 0.011, after adjustment for sex and age at T_0_). In the R pre-frontal area, the measures of NAA/Cr (2.08 ± 0.18 vs. 1.81 ± 0.18; *p* = 0.014, after adjustment for sex and age at T_0_) in subjects with non-SPG4 were significantly lower than those in SPG4 subjects. In the same area, the measures of ml/Cr were higher for non-SPG4 patients (0.57 ± 0.13 vs. 0.67 ± 0.11; *p* = 0.03, after adjustment for sex and age at T_0_).

Comparing pHSP patients and HC subjects, after adjustment for sex and age, in the L pre-central area, the pHSP patients at T_0_ showed lower Cho/Cr concentration (0.71 ± 0.11 vs. 0.79 ± 0.15; *p* = 0.012), after adjustment for sex and age at T_0_ and higher mI/Cr levels (0.62 ± 0.13 vs. 0.56 ± 0.09; *p* = 0.032). In the left pre-frontal area, the Cho/Cr values trended to be lower in the pHSP subjects compared to the HC group, but the difference was statistically at the limit of significance (0.79 ± 0.13 vs. 0.71 ± 0.11; *p* = 0.059, after adjustment for sex and age at T_0_).

In the analysis of cHSP patients vs. HC subjects, levels of NAA/Cr measured in the R pre-central area were significantly lower in cHSP than in HC (1.83 ± 0.31 vs. 2.06 ± 0.24), with strong statistical significance even after adjustment for sex and age at T_0_ (*p* = 0.005). In cHSP patients, the mean value of mI/Cr in the L pre-central area was significantly higher than in the HC group (0.70 ± 0.11 vs. 0.56 ± 0.09), with strong statistical significance after adjustment for sex and age at T_0_ (*p* = 0.001). In the R pre-frontal area, NAA/Cr was significantly lower in the cHSP patients than in the HC group (1.64 ± 0.16 vs. 1.84 ± 0.18 vs. *p* = 0.004, after adjustment for sex and age at T_0_).

Considering the clinical distinction of the patients in “pure” and complex” forms, NAA/Cr levels were higher in “pure” than in “complex” HSP subjects in the R pre-central area (1.81 ± 0.20 vs. 1.63 ± 0.26; *p* = 0.027, after adjustment for sex and age at T_0_) and in the R pre-frontal area (1.81 ± 0.14 vs. 1.64 ± 0.16; *p* = 0.021, after adjustment for sex and age at T_0_).

Examining the correlation between metabolites and the SPRS score at T_0_, we found a significant positive correlation with mI/Cr in the left pre-central area of HSP patients (*p* = 0.032, after adjustment for sex and age), while in the R and L pre-frontal areas, lower SPRS values were associated with higher NAA/Cr concentrations (after adjustment for sex and age, respectively: R, (*p* = 0.014; L, *p* = 0.005).

Regarding the correlation between metabolites and DD, a significant positive correlation was found with Cho/Cr in the L pre-central area (*p* = 0.021, after adjustment for sex and age).

### 3.2 Longitudinal analysis

The longitudinal analysis involved fewer patients (*n* = 30) due to some being lost to follow-up. A statistically significant increase in mI/Cr concentration (*p* = 0.014) was observed in the L pre-frontal area among HSP subjects at follow-up (0.72 ± 0.11) compared to baseline values of the same HSP patients (0.61 ± 0.14).

The same longitudinal analysis applied to the HC group did not show any statistically significant differences.

When comparing the variation of the main values between HSP subjects and HCs, no significant differences were found, except for higher difference from T_0_ to T_1_ in patients than in controls in the second peak of lipids. This was considered in terms of basal line variations and too influenced by artifacts to be considered a reliable result.

Clinically, the longitudinal analyses performed among HSP patients showed a significantly higher SPRS score at follow-up (18.0 ± 9.6 at baseline vs. 22.7 ± 9.7 at follow-up).

### 3.3 Decision tree method

**Figure 2:**
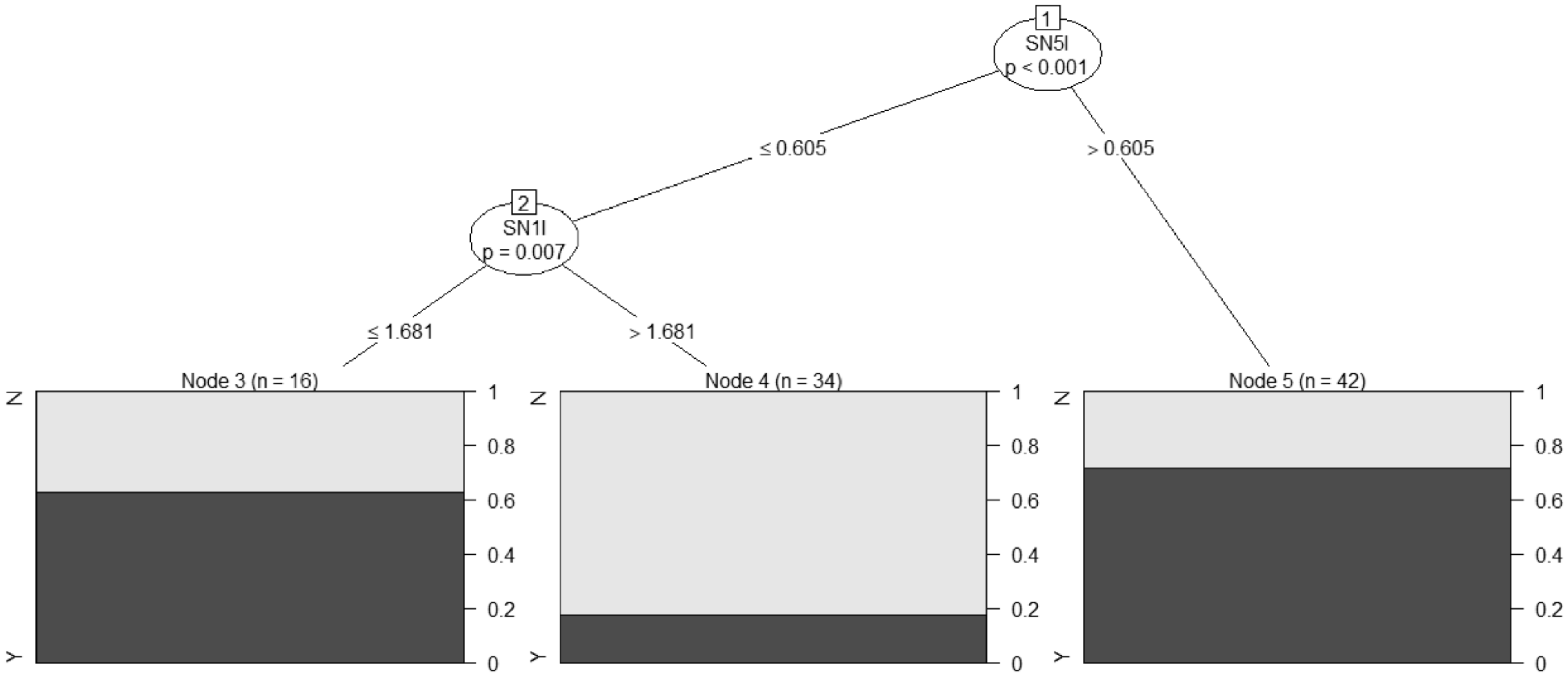
A conditional inference tree was developed on four combinations of metabolite/area, which showed statistically significant differences among HSP patients and HC: mI in the left and right pre-central areas, NAA in the right pre-frontal area, and NAA in the left pre-central area. The percentage of HSP patients correctly predicted for each node is reported in black (HC in gray). SN5I, mI in the pre-central left area; SN1I, NAA in the left pre-central area.

A conditional inference tree method was applied using the four metabolite/area combinations that showed in the present study statistically significant differences between HSP patients and HC (Figure 2): mI in the L and R pre-central areas, NAA in the R pre-frontal area, and NAA in the L pre-central area. This approach, as expected, confirmed the previously reported results.

Specifically, mI in the left pre-central area at levels >0.605 correctly classified 30 out of 42 HSP subjects, with 12 false positives (Node 5 in Figure 2). When mI in the left pre-central area was at levels less than or equal to 0.605, and NAA levels in the same area were ≤1.681, 10 HSP patients were correctly classified with six false positives (Node 3 in Figure 2).

With this decision tree method, it was possible to classify HSP subjects and controls with an overall accuracy (total number of subjects correctly classified as HSP or control) of 73.9%, a sensitivity (number of HSP patients correctly classified as HSP) of 87.0%, and a specificity (number of HC correctly classified as control) of 60.9%.

## 4 Discussion

We report metabolic changes that distinguish HSP patients from HC using 1H-MRS, sampled in MRI-appearing normal pre-central and pre-frontal cerebral regions. In the basal cross-sectional analysis (T_0_), the most relevant results comparing both groups as a whole consisted of a significant statistical reduction of NAA and an increase in mI in HSP patients. Analyzing different subgroups, non-SPG4 patients showed higher mI values and lower NAA than SPG4 patients. When dividing the phenotypes into pure and complicated, compared to HC, both showed increased mI, while only the pure form showed lower Cho, and the complicated form showed lower NAA values. Comparing these phenotypes groups, we found reduced NAA values in the complicated form. Considering the clinical performances measured with the SPRS, the scores had a directly proportional relationship with mI values and correlated inversely with NAA. Disease duration was correlated at baseline with increased Cho values in the pre-central area. The longitudinal analysis (T_1_) included only 30 patients and 30 HC from the same T_0_ group. A statistically significant increase in mI was found in HSP patients in the L pre-frontal area compared with their baseline.

In the last two decades, more than 90 SPG loci/genes have been identified. As this number is expected to grow, providing technical approaches to follow the underlying biochemical interpretation becomes fundamental. This could advance knowledge and, above all, a better monitoring response to possible therapies.

Complementary to structural MRI, the 1H-MRS allows the non-invasive *in vivo* quantification of some brain metabolites based on their characteristic magnetic resonance frequency within a magnetic field. These techniques have been widely applied to treat other neurodegenerative diseases, such as amyotrophic lateral sclerosis (ALS) (Christidi et al., 2022). In our study, cross-sectional 1H-MRS data confirm the capability of MRS to identify an HSP patient’s biochemical signature compared to HCs and to differentiate within the subgroups of patients. This leads us to believe that 1H-MRS, if correctly applied and used with adequate technical approaches, can provide elements that work as biomarkers of disease.

In a previous review, the problem of the technical differences among scientific reports on MRS and HSP was strongly emphasized, making the comparison often imprecise or impossible (Vavla et al., 2020). In particular, two fundamental aspects were considered: the different cerebral areas sampled with the single voxel method (frontal, parietal, occipital cortex, centrum semiovale, splenium of corpus callosum, corona radiata, basal ganglia, thalamus, periventricular WM, cerebellar GM, and brain stem; CSF of the lateral ventricles) and the extremely variable genetic background of the patients analyzed. Despite the extreme variability of the technical approach, the finding of a reduced NAA level in our study is frequently described in other reports on HSP (Stromillo et al., 2011). Few articles did not report such variation (see Vavla et al., 2020).

The reduction in NAA reflects a consistent degradation of function or the number of neurons in the regions involved in the motor/cortico-spinal tract system (Pizzini et al., 2003). Reduced levels of *in vivo* NAA and NAA/Cr can be observed in various neurodegenerative disorders associated with neuronal/axonal loss or compromised neuronal metabolism, highlighting the important role of 1H-MRS in diagnosis or prognosis (Rule et al., 2004; Sharma et al., 2011; Dey et al., 2023). For example, In ALS, NAA is frequently used as a surrogate marker of therapeutic efficacy on neuronal integrity (Kalra, 2019). In our study, NAA was reduced more in complicated HSP phenotypes than in pure, as reported in the literature (Stromillo et al., 2011), possibly indicating specific neuronal/axonal involvement in these cases. However, it is important to note that NAA remains an enigmatic molecule. NAA neuronal levels increase during early postnatal CNS development when it is transported from neurons to the cytoplasm of oligodendrocytes and used in fatty acid and steroid synthesis, becoming fundamental as a building block for myelin lipid synthesis. When postnatal myelination is completed, NAA likely continues to be involved in myelin lipid turnover in adults, but it also takes on other roles, such as in neuronal mitochondria bioenergy (Moffett et al., 2007).

Changes in NAA are evaluated with 1H-MRS either as an absolute concentration or as a ratio between NAA and other metabolites, almost always versus total creatine (NAA/Cr). Why is creatine used as a reference metabolite? In MRS, it represents αN-methylguanidine acetic acid, a nitrogenous organic acid that plays an important role in ATP regeneration through the Cr/phosphocreatine/creatine kinase (Cr/PCr/CK) system. Tissues such as the heart, muscle, and brain, which have high ATP demand, show elevated Cr levels. In 1H-MRS, there are three different levels of chemical shifts of Cr (at 3.029 ppm, 3.930 ppm, and 6.581–7.296 ppm), too small to be reliably distinguished at the low magnetic field (< 3 T), so it is common to describe a Cr/PCr peak at 3.07 ppm. This peak is easy to detect and has relatively constant levels in the brain under physiological conditions, making tCr a convenient internal concentration reference. However, this approach should be used with caution as Cr variability can be detected in regional changes between gray and white matter during brain development and in pathologies with impaired cell energetics (Rackayova et al., 2017). Despite these limitations, using Cr as a reference is preferable over other methods that are not easily applicable. For example, using water concentration as an internal reference can eliminate possible bias in concentrations of other brain metabolites and precisely detect tCr changes, but it is extremely time consuming and not routinely available (Kalra and Arnold, 2004). In HSP, Cr levels were analyzed as absolute concentrations with non-homogeneous results; they were reported as normal in the cortex, BG, and the CSF of the lateral ventricles, reduced in parietal and frontal WM, and increased in the parietal-occipital WM and occipital GM in one study (Vavla et al., 2020).

The other abnormal metabolic change found in our study refers to the increased value of mI in pre-central areas. These findings have also been highlighted in previous reports, but with extreme variability in terms of anatomical site, SPG type, and results, often ranging from normal to increased or decreased levels (Vavla et al., 2020). Increased mI was considered more indicative in SPG5, a form believed to be associated with inflammatory reactions (Roos et al., 2014), and in SPG2, associated with mutations in PLP1 (Svenstrup et al., 2010). It has been postulated that mI, similar to Cho, is an expression of oligodendrocytes’ activity. In HSP, the increase in mI could indicate alterations in oligodendrocyte-mediated myelination and impaired motor protein intracellular cargo transport along the CST (Blackstone, 2012). The increased mI/Cr ratio was found to be at the limits of statistical significance in SPG4 (Stromillo et al., 2011), while in our study, there is a consistent statistical difference when comparing the global groups of HSP patients and HC, non-SPG4 vs. SPG4, and complicated vs. pure forms. This indicates a close correlation between mI levels and the severity of the HSP form, as also underlined by the direct correlation between mI and clinical severity evaluated with the SPRS. It apeears that mI variations are expressions of ineffective attempts to repair the continuous, slow damage at the microstructural brain level.

Cho levels are reported as variable depending on the anatomic region sampled (Roos et al., 2014). In a previous study reporting results from only a fraction of the present cohort, we found a significant decrease in Cho in HSP patients. However, in the current analysis, we could not replicate this finding, likely due to the larger number of subjects (46 patients and HCs vs. 31 patients in the previous report) (Montanaro et al., 2020). Here we reported the only Cho variation related to some HSP subtypes (pure vs. HC) and directly correlated with disease duration at baseline, which is not easily interpretable. Choline is a constituent of the phospholipid metabolism of cell membranes, reflecting membrane turnover. A reduction in 1H-MRS Cho can be associated with various pathologic conditions and neurodegenerative diseases. It is considered an indicator of cell membrane synthesis, structural integrity, and gliosis, and could be significantly increased in regions such as the basal ganglia and the thalamus of ALS patients, probably reflecting increased astrocytosis and glial proliferation, as identified by histopathological studies (Sharma et al., 2011). In HSP, Cho has been reported as stable in some studies, decreased in others (Orthmann-Murphy et al., 2009), or even increased in some anatomical sampling (Stromillo et al., 2011; Roos et al., 2014).

Additional peaks could be detected with 1H-MRS, often associated with specific conditions. Among these is Lac, an anaerobic glycolysis product that increases when the Krebs cycle is impaired. We found no abnormal peaks, which were rarely reported by other authors (Yousem et al., 1991; Stromillo et al., 2011; Roos et al., 2014). Similarly, we did not find abnormal peaks corresponding to macromolecules. These complex molecular accumulations have a range of precession close to lactate and lipids, and can be revealed with 1H-MRS. It is normal to find them in the myelinating brain during the first years of life, while in adults, they can be associated only with pathological conditions, such as necrotic degenerative tissues or severe brain injury conditions. They are poorly identified in HSP (Yousem et al., 1991). Another group of molecules detectable with 1H-MRS is the “Glx” complex, which includes glutamate and glutamine, often almost overlapping on low magnetic field instrumentations. Along with GABA, they are considered markers of the neurotransmitter system. Both are under intense observations in ALS, as they can vary linearly with clinical severity. However, they are not routinely studied, as they need specifically dedicated acquisition, post-processing analysis, and high-field machines (at least 3.0 Tesla) to be correctly detected (Foerster et al., 2012; Sako et al., 2021).

Identifying metabolite changes in HSP does not merely involve profiling the disease but also offers insights into the neuropathology of HSP. As the lifespan of HSP patients is not particularly shorter than that of the normal population, and since they rarely die in hospitals, neuropathological reports on HSP remain rare (Behan and Maia, 1974; Bisharat-Kernizanl et al., 2018). The main pathology findings suggest the degeneration of the distal ends of axons with progression toward the cell body, which has been correlated with the “dying back” axonopathy hypothesis (Deluca et al., 2004). This is reflected by the bilateral degeneration of the CSTs, late loss of Betz cells and spinal motor neurons, symmetrical degeneration of the gracile and cuneate fasciculi, higher involvement at the thoracic level progressing toward the cervical regions up to the bulbar nuclei, and bilateral loss of fibers in the spinocerebellar tracts (Behan and Maia, 1974; Deluca et al., 2004; Wagner et al., 2019). The preferential involvement of the distal ends of the longest motor and sensory axons suggests a generalized failure of the cells to maintain the vitality of these long axons as a possible pathogenetic mechanism, which contrasts with the idea of HSP as a disease affecting a single neuronal system (e.g., the motor system).

This distinction differentiates HSP from ALS, where the hallmark histopathological features are motor neuron loss in the motor cortex and the spinal cord, and/or CST degeneration (Mazón et al., 2018). These distinct disease processes characterizing HSP and ALS converge in a “common route,” involving motor neurons in ALS at the beginning of the disease and in HSP in the advanced stages, resulting for both the diseases in a CST damage (Denora et al., 2016).

Another final route common to HSP and ALS is the fundamental observation that modern clinical and paraclinical studies show that the brain structures involved are not restricted to the motor/CTS complex but extend to the whole brain (Aghakhanyan et al., 2014; Agosta et al., 2015). This can also be observed in post-mortem findings (Garaci et al., 2014; Agosta et al., 2015; Boutry et al., 2019; Coarelli et al., 2019), supporting the definition of HSP as a “*metasyndromic umbrella*” characterized by a common final pathological event sustained by many diverse metabolic mechanisms (Martinuzzi et al., 2016; Faber et al., 2018), also involving infra-tentorial structures, particularly the cerebellum (Lindig et al., 2015; Lupo et al., 2020; Servelhere et al., 2021).

In line with the view of wider brain involvement in HSP, the frontal lobe is particularly observed, especially for cognitive impairment in almost all HSP patients, including pure forms (Duning et al., 2010). We observed a significant reduction of NAA in the pre-frontal samplings comparing the whole HSP group to HC, in non-SPG4 vs. SPG4 groups, and complicated vs. pure phenotypes, in which there was also an increase in mI. Interestingly, in the longitudinal analysis, the only statistically significant data in the HSP patients related to an increased mI content in pre-frontal sampling compared to basal levels.

The involvement of the frontal and pre-frontal regions has already been reported to correlate with the reduction of NAA and the cognitive/behavioral symptoms in some forms of HSP longitudinal assessment (Dreha-Kulaczewski et al., 2006). In another study, a close correlation was observed between the physiologic aspects of the role of the pre-frontal regions in facial expression processes (Balconi and Bortolotti, 2013).

It is likely that the involvement of the pre-frontal areas should be considered among the altered executive processing of motor function in these patients, similar to ALS patients (Quinn et al., 2012). However, targeted tests underline subtle cognitive deficits in both pure and complicated HSP phenotypes, such as in verbal abstract thinking and verbal fluency, which is suggestive of frontal lobe dysfunction (Erichsen et al., 2009; Liguori et al., 2014; Utz et al., 2022).

The longitudinal MRS results in the MRS samplings areas are novel and have not been reported before, even though studies on HSP follow-up are rare, especially because HSPs are very slowly progressive disorders with subtle longitudinal changes (da Graça et al., 2018). This translates into conflicting results, sometimes leading to surprising, hardly generalizable data (Montanaro et al., 2020), often derived from considerations from single case studies (Dreha-Kulaczewski et al., 2006) or concerning only specific aspects of HSP, such as the progression of the corpus callosum thinning (França et al., 2012).

The loss of function or the depletion of neurons and glial reaction to brain damage, probably represented in our study by decreased NAA and increased mI observed in 1H-MRS, are not sufficient to fully describe the neuropathological and biochemical processes developing during the natural history of any genetic HSP variation. The axonopathy observed in HSP is associated with axonal loss and decreased spinal cord caliber from the lumbar level to the medulla (Deluca et al., 2004), mainly sustained by the focal swelling of axons with the accumulation of organelles and intermediate filaments compatible with impaired retrograde transport (Tarrade et al., 2006; Salinas et al., 2008; Shribman et al., 2019). Axons in the efferent CST and afferent sensory tracts are very long and represent the extensions of neurons that need multiple cellular components to survive, including structural proteins, organelles, membrane trafficking, metabolic support, signaling, and degradation machinery, all immersed in an unstable equilibrium of homeostasis of energy, lipids, and proteins (Smith et al., 2023). In this scenario, many recent scientific reports address the variability of the genetic background of HSPs. In most of them, it is possible to identify a specific metabolic defect: many genes implicated in HSP are crucial to molecular trafficking, transport, and energy metabolism along the exceptionally long axons of the CST and sensory tracts. It appears likely that their terminal ends are particularly vulnerable (Deluca et al., 2004; Blackstone, 2018). For example, in SPG4, a deficit of the protein spastin causes impaired microtubule maintenance, thereby affecting the microtubule-based transport of mitochondria and peroxisomes, resulting in the swelling of neurons and axonal, impaired neurite growth, and leading to axon degeneration (Wharton et al., 2003; Wang et al., 2023). In SPG7, the loss of paraplegin, localized in the inner mitochondrial membrane, leads to mitochondrial dysfunction (McKay and Tkáč, 2016). In SPG3A, atlastin deficit impairs endoplasmic reticulum shaping (Candia et al., 2023), while in SPG5, the damage of the CYP7B1 gene, involved in cholesterol metabolism, results in the raised levels of toxic 27-hydroxycholesterol and 25-hydroxycholesterol in plasma and cerebrospinal fluid (Roos et al., 2014), and in SPG11, spatacsin-deficient cells show impaired transport of synaptic vesicles and neurite growth (Mackay-Sim, 2021; Van de Vondel et al., 2024). The consequence is that many fundamental cellular functions involving the Golgi apparatus, mitochondria, and endoplasmic reticulum (ER) are affected, each playing a possible role in the pathogenesis of HSPs. Among these, the ER is emerging as key for synthesizing, metabolizing, and distributing proteins, lipids, and sterols, employing both vesicular and non-vesicular mechanisms (Blackstone, 2012; Rinaldi et al., 2015; Darios et al., 2020). Unfortunately, similarly to other cellular organelles, any MRI-corresponding variation at this level cannot be identified, even with ultra-high fields. Only indirect elements indicative of a cellular defect can be described. For instance, in our study, we had the technical possibility with 1H-MRS to identify two peaks referring to lipids, highlighting possible variations in HSP in which lipids are involved, but their low levels were easily confounded with the baseline and consequently often not acceptable as a true statistical variation.

Components with a fundamental role in lipid pathway alteration are the so-called lipid “droplets” (LD). They are storage organelles made of a core of neutral lipids surrounded by a monolayer of phospholipids, whose formation occurs in the membrane of the ER (Li, 2021; Tadepalle and Rugarli, 2021). Their activity is dynamically involved in lipid metabolism and energy homeostasis, and they play a key role in mitochondria, lysosomes, synaptic function, and myelin and axonal integrity maintenance. The hypothesis of a central role in motor neuron dysfunction in HSP is reasonable (Denton et al., 2018). Mutations in cytochrome P450-7B1 (CYP7B1) in SPG5 patients, loss of spatacsin due to mutations in SPG11, mutations in DDHD1 in SPG28 or DDHD2 in SGP54 (the latter with a well-known 1H-MRS abnormal lipid peak in the basal ganglia and the thalamus) (Schuurs-Hoeijmakers et al., 2012; Doi et al., 2014; Zaidi et al., 2019), and sphingolipids derived from the sphingosine backbone synthesized in the ER and the Golgi apparatus, altered in SPG35 (Darios et al., 2020), are only some examples of lipid involvement in HSP. In recent years, lipids have attracted enormous interest in connection with modern approaches to therapy in laboratory models (Napoli et al., 2019; Mou et al., 2020), so one of the challenges ahead is to focus on 1H-MRS for the correct detection of lipid macromolecules.

In this study, we attempted to combine the various metabolites detected with 1H-MRS to obtain a decision-guided analysis to develop a single patient data interpretation (Figure 2). The combination of more than one biomarker considered with specific algorithms to distinguish patients from HC is frequently reported using advanced MRI quantitative methods in neurodegenerative disease, for example, analyzing DTI and brain morphometric data in ALS (Foerster et al., 2014).

To the best of our knowledge, this is the first study metabolic 1H-MRS variations in a group of HSP patients interpolated among them to extrapolate a decision-guided analysis. The goal was to discriminate single patients against normal subjects, but the decision tree model described in this study is not endowed with statistically predictive power because of the limited number of subjects, and this did not allow a validation protocol based on the database splitting into a training and a test set. However, the model can provide insight into some potential metabolic patterns for the diagnosis of HSP subjects, considering the possible future approach with artificial intelligence and machine learning, including deep learning and other random forest algorithms, as it is already underway for other diseases or with other types of parameters (Christidi et al., 2022; Awuah et al., 2024).

With our current data processing, we can analyze the potential of considering 1H-MRS as a biomarker for HSP. The concept of a “biomarker” has often been discussed in the context of neurodegenerative disease. It should be “… *a subrogated feature and parameter extracted from medical data that gives quantitative information on the regional distribution and magnitude of the depicted characteristic*” (Mazón et al., 2018). Moreover, it should have characteristics of objectivity, not being influenced by the placebo effect, being specific for a given disease, and being sensitive to variations in time due to the natural disease history or response to therapy. Ideally, this would be represented by the sampling of only one catabolite circulating in the serum, but the brain is protected from the rest of the body and circulating blood by the blood–brain barrier (Krumm et al., 2024). Sampling CSF has limitations as it requires a lumbar puncture, which is invasive and justified only for diagnostic purposes, not for repeated follow-up or screening in the general population, and does not provide any map of regional brain involvement (McKay and Tkáč, 2016). This highlights the encouraging role of methods such as 1H-MRS and decision-guided analysis, as proposed in the present study.

### Limitations

Besides those already reported, limitations for each argument can be listed here. First of all, various biases, both at the acquisition and in the post-processing phase, are unfortunately critical, as in other 1H-MRS studies. Different acquisition parameters, compared to other studies, depend on the low magnetic field applied (1.5 T) and the software for processing methods, which are provided directly by the vendor and not verified using other software. A more strictly technical aspect concerns the fact that a 1H-MRS spectrum is represented by the height and the subtended area of the peaks and curves of each metabolite (Pioro, 2000). The approach to these values could be bivalent, as sometimes one cannot exclude or consider the other. However, it is fundamental to analyze all subjects, patients, and HC similarly (Han and Ma, 2010). The extension and the type of brain tissue sampling is another limitation to be considered, as often it can include white and gray matter (Moffett et al., 2007): it is possible to normalize the single voxel for each component (Ellis et al., 1998), but as we applied the identical approach in our study to both patients and HC, this should have minimized the bias.

The problem of artifacts, which is very important for any qualitative and quantitative imaging and advanced method, is fundamental in MRI. In particular, in 1H-MRS, improper water suppression, chemical shift artifact/misregistration, field inhomogeneity, altered T1 and T2 relaxation times, and more, can lead to wrong spectra interpretation, not forgetting the variability of vendor-supplied platforms (Moss et al., 2019).

The limited number of subjects, the mixed genetic types, and the short duration of the longitudinal sampling are other important limitations of this study and other studies. These factors have been well known since the initial reports on MRI applications in HSP (Yousem et al., 1991). They are expected when we approach rare diseases (Martinuzzi et al., 2016), not forgetting the difficult management for such clinical patients and families (Fink, 2002). To partially overcome these difficulties, as other authors and we proposed (Montanaro et al., 2020), there is a need for *“…creating a networking study group to focus on the neuroimaging approach”* (Vavla et al., 2020).

In our cohort, we included a few patients with non-defined genetic SPG (5 out of 46 patients), but they fulfilled the criteria for a clinical diagnosis of HSP. This is a well-known problem in considering rare diseases, as exemplified in a recent report of Asian HSP patients, where 108 patients out of 270 did not have a molecular diagnosis, even after applying the most modern genetic investigations (Cao et al., 2024).

An apparent problem could be the unilaterality or asymmetry of some of our results. This is not correlated with specific elements in our study, but it is not surprising as morphologic and morphometric alterations could also be unilateral or asymmetric, and at an individual level, asymmetrical symptoms and brain pathology are often reported (Yousem et al., 1991; Bede and Hardiman, 2014).

Some authors propose the use of high or ultra-high magnetic fields. There is certainly an advantage in spectral resolution when using the highest possible fields, but presently, there are still management problems and limited evidence of real advantages from using 7.0 Tesla (McKay and Tkáč, 2016; Atassi et al., 2017; Cheong et al., 2017, 2019). The higher purchase and operating costs of these machines (Kalra, 2019) and the linearity of the artifact, which always lurks and increases when managing patients who are not completely collaborative, should be considered (Liserre et al., 2021).

Some previous reports indicated other MRI elements indicative of HSP, from simple semeiotic MRI signs, which are extremely useful, especially in childhood (Dosi et al., 2021), to more advanced quantitative approaches (Aghakhanyan et al., 2014; Agosta et al., 2015; Chojdak-Łukasiewicz et al., 2023). However, the attempt at a biochemical approach, as in our study, is captivating from the pathophysiologic point of view and from the perspective of therapy development. It does not exclude the possibility of more consistent future integration among the various methods, such as PET with MRI (McMackin et al., 2023).

## 5 Conclusion

This study highlights the importance of MRS in revealing variations in HSP through two fundamental metabolites: NAA (a marker of neuronal integrity) and mI (described as an astroglial marker). There is a strong statistical association between increased mI and/or decreased NAA, observed not only by comparing the whole group of HSP patients and HC but also by examining various subgroups. These subgroups include genetic forms (no-SPG4 vs. SPG4), phenotype presentation (complex vs. pure forms), clinical performances measured with the SPRS (scores directly proportional to mI values and inversely proportional to NAA), and longitudinal variations (increased mI in follow-up compared to baseline values in patients), all indicating a close correlation with the severity of HSP.

By combining the NAA and mI Cr-ratio, we showed the possibility of distinguishing HSP patients from HC with a decision tree model analysis. Although these last data need a validation step (blinded assignment to the HSP vs. HC group), by including other MRS metabolites in the model (for example, lipids combined with concentrations of NAA, mI, and Cho), MRS could contribute further as a specific biomarker. It could be applied as a translational method considering recent advancements in cell biology and laboratory animal models of HSP (Damiani et al., 2024).

The lack of a cure for HSP is well known, with current treatments aimed at symptomatic relief (Synofzik and Schüle, 2017). However, the rapid increase in knowledge of causal genes is leading to the identification of a large number of potential pathogenetic mechanisms that can offer new treatment opportunities, such as targeted molecular therapies (Shribman et al., 2019). The increasingly pressing necessity of appropriate biomarkers is becoming more integrated into clinical practice (Gumeni et al., 2021; Li, 2021; McMackin et al., 2023; Siow et al., 2023; Hörner et al., 2024; Krumm et al., 2024). The statistically consistent data reported in this study, if confirmed by applying modern technical MRS approaches (absolute metabolite concentrations and quantifiable cortical–subcortical WM samplings along the CST regions with high magnetic field strength equipment scans), can become a starting point for investigating new specific metabolites within the 1H-MRS spectrum.

## Data availability statement

The raw data supporting the conclusions of this article will be made available by the authors, without undue reservation.

## Ethics statement

The studies involving humans were approved by the Ethics Committee (# 06/2020CESC). The studies were conducted in accordance with the local legislation and institutional requirements. The participants provided their written informed consent to participate in this study.

## Author contributions

DM: Conceptualization, Methodology, Writing – original draft, Writing – review & editing, Data curation. MV: Conceptualization, Data curation, Writing – review & editing. FF: Data curation, Methodology, Writing – original draft, Writing – review & editing. AC: Data curation, Writing – original draft, Writing – review & editing. AB: Data curation, Investigation, Writing – review & editing. RP: Data curation, Writing – review & editing. CS: Data curation, Writing – review & editing. AM: Conceptualization, Supervision, Writing – original draft, Writing – review & editing.
