# Supplementary material for: Metabolite profile in hereditary spastic paraplegia analyzed using magnetic resonance spectroscopy: a cross-sectional analysis in a longitudinal study
Source: Front Neurosci. 2024 Aug 13;18:1416093. doi: 10.3389/fnins.2024.1416093 (PMC11347332; doi:10.3389/fnins.2024.1416093)
Supplement: Supplementary file 1 [file Table_1.DOCX]

| **Table 1: Demographic, clinical and genetic data of HSP patients and healthy control group** | | |
| --- | --- | --- |
|  | **HSP patients**  **N=46** | **Healthy controls**  **N=46** |
| Age at first MRI (years) | 41.5 ± 15.9 | 42.2±12.2 |
| Gender (female) | 25 F, 21 M | 32 F, 14 M |
| Disease duration (years) | 27 ± 20 |  |
| Disease onset (years) | 15 ± 13 |  |
| **Spastic gate loci** |  |  |
| SPG4 | 18 |  |
| SPG5 | 5 |  |
| SPG11 | 2 |  |
| SPG30 | 1 |  |
| SPG3 | 6 |  |
| SPG8 | 2 |  |
| SPG7 | 3 |  |
| SPG72 | 2 |  |
| SPG10 | 1 |  |
| SPG31 | 1 |  |
| SPG? | 5 |  |
| Pure/complicated | 27/14 |  |
